# Cocaine/levamisole-induced systemic vasculitis with retiform purpura and pauci-immune glomerulonephritis

**DOI:** 10.1590/1414-431X20165244

**Published:** 2016-04-26

**Authors:** F.V. Veronese, R.S.O. Dode, M. Friderichs, G.G. Thomé, D.R. da Silva, P.G. Schaefer, V.C. Sebben, A.R. Nicolella, E.J.G. Barros

**Affiliations:** 1 Serviço de Nefrologia, Hospital de Clínicas de Porto Alegre, Universidade Federal do Rio Grande do Sul, Porto Alegre, RS, Brasil; 2 Serviço de Patologia, Hospital de Clínicas de Porto Alegre, Universidade Federal do Rio Grande do Sul, Porto Alegre, RS, Brasil; 3 Centro de Informação Toxicológica, Fundação Estadual de Produção e Pesquisa em Saúde, Porto Alegre, RS, Brasil

**Keywords:** Cocaine, Levamisole, Systemic vasculitis, ANCA, Retiform purpura, Crescentic glomerulonephritis

## Abstract

Levamisole has been increasingly used as an adulterant of cocaine in recent years, emerging as a public health challenge worldwide. Levamisole-associated toxicity manifests clinically as a systemic vasculitis, consisting of cutaneous, hematological, and renal lesions, among others. Purpura retiform, cutaneous necrosis, intravascular thrombosis, neutropenia, and less commonly crescentic nephritis have been described in association with anti-neutrophil cytoplasmic antibodies (ANCAs) and other autoantibodies. Here we report the case of a 49-year-old male who was a chronic cocaine user, and who presented spontaneous weight loss, arthralgia, and 3 weeks before admission purpuric skin lesions in the earlobes and in the anterior thighs. His laboratory tests on admission showed serum creatinine of 4.56 mg/dL, white blood count 3,800/μL, hemoglobin 7.3 g/dL, urinalysis with 51 white blood cells/μL and 960 red blood cells/μL, and urine protein-to-creatinine ratio 1.20. Serum ANCA testing was positive (>1:320), as well as serum anti-myeloperoxidase and anti-proteinase 3 antibodies. Urine toxicology screen was positive for cocaine and levamisole, with 62.8% of cocaine, 32.2% of levamisole, and 5% of an unidentified substance. Skin and renal biopsies were diagnostic for leukocytoclastic vasculitis and pauci-immune crescentic glomerulonephritis, respectively. The patient showed a good clinical response to cocaine abstinence, and use of corticosteroids and intravenous cyclophosphamide. Last serum creatinine was 1.97 mg/dL, white blood cell count 7,420/μL, and hemoglobin level 10.8 g/dL. In levamisole-induced systemic vasculitis, the early institution of cocaine abstinence, concomitant with the use of immunosuppressive drugs in severe cases, may prevent permanent end organ damage and associate with better clinical outcomes.

## Introduction

Illicit drug use and dependence directly accounted for 20 million disability-adjusted life years in 2010 and 0.8% of the global burden of disease worldwide, mostly due to opioid dependence (1). In South America, cocaine consumption and trafficking have become more prominent, particularly in Brazil (2). Levamisole, a drug indicated for the treatment of parasitic diseases and as an immunomodulatory agent, is an increasingly common adulterant in cocaine. It is estimated that over 70% of cocaine currently consumed in the United States contains levamisole (3). Due to its association with serious adverse effects, such as agranulocytosis and vasculitis, levamisole was withdrawn from use in human medicine, but is still widely available for veterinary use (2,3).

The first reported associations of levamisole use with cutaneous leukocytoclastic vasculitis (4) and nephropathy (5) were published in 1978. Since 2009, there have been successive reports of systemic vasculitis in users of levamisole-contaminated cocaine, a condition characterized by retiform purpura, neutropenia, intravascular thrombosis, and pauci-immune crescentic glomerulonephritis in the presence of anti-neutrophil cytoplasmic antibodies (ANCAs) and other autoantibodies (6-10). The growing incidence of cocaine/levamisole-associated vasculitis has become a major public health concern worldwide (2,11,12). Discontinuation of the offending drugs plays a critical role in the treatment of these patients, and depending on the severity of the clinical presentation, immunosuppressive drugs have been used as well (6-8,10).

Here we describe a patient with ANCA-positive systemic vasculitis, manifested as cutaneous retiform purpura, leukopenia, and crescentic glomerulonephritis, in whom cocaine adulterated with levamisole was detected in urine.

## Case Report

**Figure 1:**
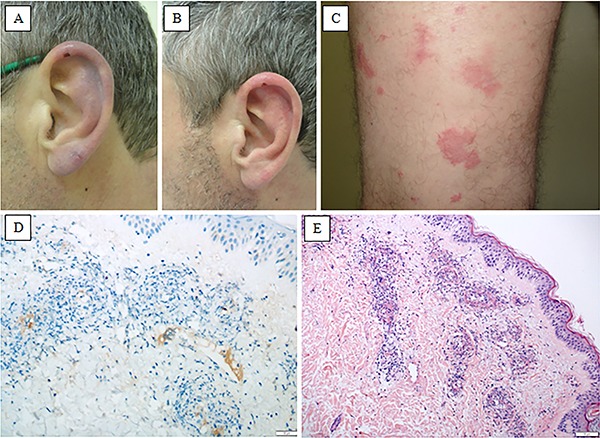
Skin lesions: *A*, Retiform purpura with a small area of necrosis in the right earlobe (pre-treatment). *B*, Residual skin lesions in the right earlobe after 3 weeks of immunosuppressive treatment. *C*, Purpuric violaceous lesions with surrounding erythema in the lower limb. Skin biopsy: *D*, Immunohistochemistry with anti-CD61 antibody showing positive staining for thrombi inside the vascular lumen, with surrounding inflammation of the vessel wall (magnification 100×). *E*, Small vessel vasculitis with neutrophilic inflammation and leukocytoclasia (H&E, magnification 100×).

A 49-year-old white male presented to the emergency department with a chief complaint of spontaneous weight loss (20 kg in 1 year) and arthralgia. He reported development of erythematous lesions on the earlobes and anterior surface of the thighs 3 weeks before presentation. Medical history was positive for arterial hypertension that was diagnosed 2 years before but not treated, and alcohol and cocaine dependence. The patient was receiving psychiatric care for depression. Current medications included 1 mg/day risperidone, 40 mg/day fluoxetine, and 500 mg/day sodium valproate, the latter for seizures during alcohol and cocaine withdrawal. He denied any prior kidney conditions, and his baseline serum creatinine measured 1 year before was 0.8 mg/dL. Physical examination revealed erythematous, slightly hypochromic skin lesions on the anterior and posterior surfaces of the thighs and flanks bilaterally, as well as edema and purpuric areas with foci of central necrosis. The auricula was edematous and purpuric, with focal necrosis, as shown in Figure 1.

Laboratory tests on admission were as follows: urinalysis with 51 leukocytes/μL, 960 erythrocytes/μL, spot urine protein-to-creatinine ratio 1.20, serum creatinine 4.56 mg/dL, hemoglobin 7.3 g/dL, platelets 290,000/μL, WBC 3,800/μL, and serum albumin 4.1 g/dL. Complement levels were within normal limits (C3, 89 mg/dL; C4, 14 mg/dL). Anti-nuclear and anti-dsDNA antibodies, lupus anticoagulant, rheumatoid factor, cryoglobulins, and HBV, HCV, and HIV serologies were negative. ANCA testing was positive (titers >1:320), with anti-myeloperoxidase (anti-MPO) antibody 109 IU/mL (positive if >5 IU/mL) and anti-proteinase 3 (anti-PR3) antibody 35 IU/mL (positive if >10 IU/mL). Renal ultrasonography findings were normal.

**Figure 2:**
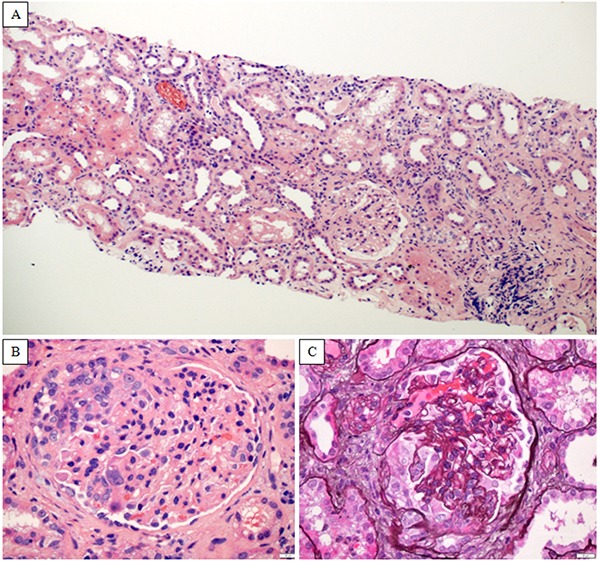
Kidney biopsy: *A*, Chronic tubulointerstitial inflammatory infiltrate composed mainly by lymphomononuclear cells (H&E, 100× magnification). *B*, The glomerulus exhibits a cellular crescent and mesangial hypercellularity (H&E, 400× magnification). *C*, Multifocal rupture of the glomerular basement membrane, with a cellular crescent in the Bowman’s space (methenamine silver, 400× magnification).

Skin biopsy revealed a neutrophilic vasculitis in small vessels with eosinophils, leukocytoclasia, and fibrinoid necrosis (Figure 1). Skin immunofluorescence showed focal and granular deposits of C3 in venules. There were a total of twenty-five glomeruli in kidney biopsy, with cellular crescents and intra-glomerular necrosis in eight. There were no globally sclerosed glomeruli. Podocyte hypertrophy, focal mesangiolysis, a diffuse and chronic inflammatory infiltrate in the tubulointerstitium, and interstitial fibrosis and tubular atrophy in 10% of total cortical area were also observed (Figure 2). Immunofluorescence findings revealed no deposits of IgG, IgM, IgA, C1q, C3, fibrinogen, kappa and lambda, which was consistent with a pauci-immune crescentic glomerulonephritis. The findings of retiform purpura, crescentic glomerulonephritis, and positive anti-MPO and anti-PR3 antibodies were compatible with exposure to levamisole-contaminated cocaine. Pulse corticosteroid therapy was instituted with intravenous methylprednisolone, 500 mg/day for 3 days. During his hospital stay, the patient exhibited a recurrence of elevated creatinine and onset of new cutaneous lesions. A second methylprednisolone pulse therapy was performed (1 g/day for 3 days) and cyclophosphamide 1000 mg *iv* was administered, which were followed by an improvement of cutaneous lesions and renal function. The patient was discharged on 60 mg/day prednisone, with a plan to receive monthly *iv* cyclophosphamide pulse therapy depending on clinical response. Guidance was provided on the importance of continued psychiatric care and abstinence from cocaine.

One week after discharge, the patient returned asymptomatic but reporting a relapse of cocaine use. A sample of cocaine powder used by the patient was sent to the Rio Grande do Sul State Poison Control Center for testing to confirm presence of cocaine and levamisole. Serial urine samples were collected for an immunochromatographic drug screen test (Abon^¯^, Biopharm, China), and confirmatory testing was performed by gas chromatography-mass spectrometry (GC/MS) in an Agilent^¯^ 7890A/5975C system (USA). Urine toxicology screen was positive for cocaine and levamisole, and the percentage of each compound measured in the first cocaine powder sample was 62.8% of cocaine, 32.2% of levamisole, and 5% of an unidentified substance.

**Figure 3:**
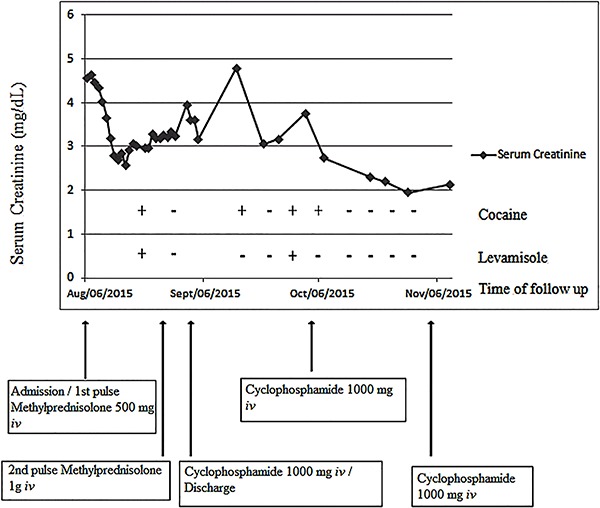
Evolution of renal function over 3 months of follow-up and its relation to urine toxicology for cocaine and levamisole, and to therapeutic interventions (methylprednisolone and cyclophosphamide intravenous (*iv*) pulses).

As there had been no significant improvement in renal function, the decision was made to continue immunosuppressive therapy and intensify psychiatric follow-up. One month after hospital discharge, the patient reported abstinence from cocaine, which was confirmed by negative urine samples for cocaine or levamisole, and exhibited progressive improvement of renal function (Figure 3). On January 2016, in the last follow-up visit, his blood pressure was 130/80 mmHg, he had a weight gain of 8 kg, and laboratory tests showed serum creatinine of 1.97 mg/dL, urinalysis with 14 leukocytes/μL, 12 erythrocytes/μL, and urine protein-to-creatinine ratio of 0.34, as presented in Table 1. ANCA titers had decreased to 1:160.

**Table:**
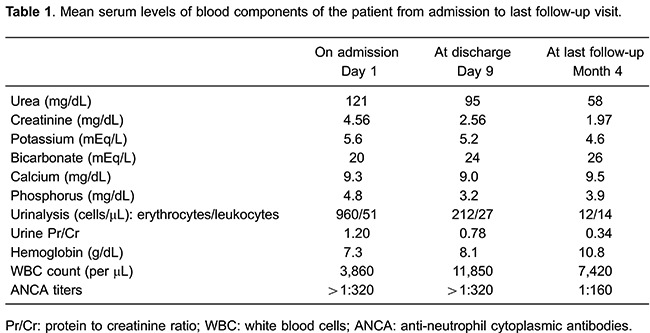

## Discussion

To the best of our knowledge, this is the first report of a Brazilian patient with levamisole-induced systemic vasculitis presenting with crescentic glomerulonephritis and severe acute renal failure, and with documented positive toxicology for cocaine and levamisole in urine samples.

From a pharmacological standpoint, cocaine increases dopamine concentrations in the synaptic cleft by inhibiting its reuptake, while levamisole, a nicotinic antagonist, releases neuronal glutamate, thus potentiating the dopaminergic effect of cocaine (12). These central and peripheral effects act synergistically to enhance cocaine addiction. As levamisole contains reactive thiol groups in its structure, it behaves as a hapten, thus triggering immune responses that promote dendritic cell maturation, proinflammatory cytokine release, autoantibody production, and cytotoxicity (13,14). These effects of levamisole cause vasculitis, necrosis, and intravascular thrombosis in several organs and tissues, such as the skin, hematopoietic system, brain, and kidneys. Renal injury also occurs as a result of the nephrotoxic effects of cocaine, which include changes in intrarenal hemodynamics, oxidative stress, extracellular matrix synthesis and degradation, and renal atherogenesis (6,9,10,15,16).

Levamisole-induced vasculitis is a diagnosis of exclusion. It should be considered in any patient with a history of cocaine use who present with the tetrad of retiform purpura involving the ear and nose, arthralgia, neutropenia, and high-titer ANCA positivity (17). As reviewed by Carlson et al. (10), three serologic profiles have been described in levamisole-induced vasculitis: no circulating autoantibodies in those with organ-limited disease, positive MPO and PR3 antibodies in patients with necrotizing systemic vasculitis, or positive cANCA and PR3 antibodies in cocaine-induced midline destructive lesions. Other autoantibodies are commonly detected, such as antinuclear, anti-dsDNA, anticardiolipin, and antihuman neutrophil elastase antibodies, as well as lupus anticoagulant (6,8,10,15,17). In a study by McGrath et al. (6) of 30 patients exposed to cocaine/levamisole, the most prevalent manifestations were arthralgia (83%), cutaneous lesions (61%), and nonspecific symptoms such as fever, weight loss, fatigue, and myalgia (72%). Nearly half of patients (44%) presented with renal injury. All cases were ANCA-positive at high titers. All had detectable anti-MPO and 50% were positive for anti-PR3 antibodies. A review of levamisole-induced leukocytoclastic vasculitis by Arora et al. (8) and later reports of patients with cutaneous lesions and/or crescentic glomerulonephritis (6,10,17) provide overlapping descriptions of clinical and laboratory findings, and skin and renal histopathology.

The most common skin biopsy findings in levamisole-associated vasculitis are intravascular thrombosis and/or leukocytoclastic vasculitis with perivascular lymphocytic infiltration, thrombotic microangiopathy, panniculitis, and/or necrosis (7,8,17). Schmoeller et al. (18) reported a Brazilian chronic cocaine user who presented skin necrosis, positive perinuclear ANCA and anti-phospholipid antibodies. In skin biopsy, there was thrombosis of small vessels in the epidermis and upper dermis, but no evidence of vasculitis. The authors did not mention renal involvement.

In most published series, renal biopsies obtained from patients with acute kidney injury reveal a focal, segmental, necrotizing glomerulonephritis with cellular crescent formation, diffuse inflammatory infiltrates, and paucity or absence of immune deposits on immunofluorescence (6,9,10,15,19). If diagnosis and treatment of crescentic nephritis are delayed, biopsy reveals fibrous crescents, interstitial fibrosis, and tubular atrophy, confirming the potential of levamisole to induce chronic nephropathy, with progression to end-stage renal disease requiring renal replacement therapy (10,15).

The mainstays of treatment of cocaine/levamisole-associated systemic vasculitis are immediate cessation of drug exposure, blood pressure management, and general supportive care. Relapse of adulterated cocaine use after initial withdrawal may lead to recurrence of vasculitis (16). Therefore, health care should focus on strategies to ensure adherence to abstinence from cocaine, preventing acquisition and use of the drug after diagnosis (2,16). Additional measures can include immunosuppressive therapy, depending on disease severity. However, the efficacy of immunosuppression and the optimal immunosuppressive regimen remain unclear, as this practice is based on experience with a limited number of patients (6,8-10,12,15). Pulse therapy with *iv* methylprednisolone followed by oral prednisone, combined with oral or *iv* cyclophosphamide and occasionally plasmapheresis, have been employed based on analogy with strategies for management of primary ANCA-associated vasculitis. The response to treatment of cutaneous lesions has been widely variable, regardless of the presence of vasculitis, thrombosis, or necrosis. Discontinuation of levamisole exposure and/or institution of immunosuppressive therapy may lead to spontaneous resolution of symptoms, rapid clinical response in less than a week, or gradual improvement up to 3 months after treatment (8).

Experience with immunosuppressive regimens in crescentic glomerulonephritis is quite limited due to the low prevalence of this condition. Reported outcomes have ranged from complete recovery of renal function, through partial response, to progression to chronic kidney disease requiring renal replacement therapy (6,10). In the case reported herein, our patient had a partial response to immunosuppressive therapy, with resolution of cutaneous lesions and improvement of renal function, especially after he achieved abstinence from adulterated cocaine.

The short elimination half-lives of cocaine and levamisole (0.7-1.5 and 5-6 h, respectively) hinder detection of these substances in body fluids (20). Levamisole can be detected up to 3 days after exposure, particularly on GC/MS testing (21). Therefore, the time to urine drug testing is critical for confirming recent exposure, as the majority of cocaine-dependent individuals are unable to remain abstinent (16).

The growing incidence of levamisole-contaminated cocaine use should heighten the index of suspicion for the potentially serious toxic effects of this harmful combination. In a patient with cutaneous lesions, neutropenia and/or glomerulonephritis, and a positive ANCA test, a search for clinical and laboratory evidence of systemic vasculitis and urine toxicology screening for these agents are mandatory. Skin and renal biopsies can confirm the presence of necrotizing vasculitis. In addition to abstinence from drugs, early institution of immunosuppressive therapy may lead to better clinical outcomes. Prospective studies with larger samples are warranted to evaluate this strategy.
